# Going Local to Global through Technology-Needs Assessment and Development of a Virtual Arctic Youth Wellbeing Network

**DOI:** 10.3390/ijerph192013290

**Published:** 2022-10-14

**Authors:** Allison Crawford, Brittany Graham, Arnârak Bloch, Alexis Bornyk, Selma Ford, David Mastey, Melody Teddy, Christina Viskum Lytken Larsen

**Affiliations:** 1 Virtual Care and Psychiatry Outreach, Centre for Addiction and Mental Health (CAMH), 60 White Squirrel Way, Office 233, Toronto, ON M6J 1H4, Canada; 2 Department of Psychiatry, University of Toronto, Toronto, ON M5T 1R8, Canada; 3 Public Health and Preventive Medicine, McMaster University, Hamilton, ON L8P 1H6, Canada; 4 Innuttaasut Peqqissusiannik Ilisimatusarfik, Center for Public Health in Greenland, University of Southern Denmark, 1399 Copenhagen, Denmark; 5 Inuit Circumpolar Council, Ottawa, ON K1P 5E7, Canada; 6 Arctic Youth Network

**Keywords:** circumpolar peoples, youth well-being, culture, mental health, indigenous health

## Abstract

Strengths-based approaches to suicide prevention and life promotion in circumpolar regions must engage youth participation and leadership given the impact of suicide on this demographic. We describe the development of a youth-engaged community of practice (CoP) across circumpolar regions, and adaptations to the ECHO model as a foundation for this virtual CoP. We describe youth priorities for learning in the area of mental health and wellbeing, identified through a learning needs assessment. A curriculum was developed to address key areas of interest, including: cultural approaches to mental wellbeing; language-based approaches to mental wellbeing; resilience; government and policy; and suicide prevention. We describe steps taken to adapt the ECHO model, and to introduce Indigenous pedagogical and knowledge sharing approaches into the CoP in order to meet youth learning interests. We conclude that this virtual CoP was a feasible way to create a learning community, and suggest that a priority future direction will be to evaluate the impacts of this virtual CoP on youth engagement, satisfaction and learning.

## 1. Introduction

Youth are both invigorating and challenging approaches to mental health and wellbeing through active participation in community-based approaches to mental health research, practice and education [1,2]. In no area is this revitalization more apparent, and more necessary, than in health and wellbeing efforts, in which Indigenous youth in particular are providing leadership [3,4,5]. This is a vital and strengths-based response to some of the challenges faced by many Indigenous youth and communities, including in suicide prevention and life promotion.

Rates of suicide are elevated among many Indigenous communities globally [6,7], including high rates of suicide within many circumpolar communities, particularly among youth [8]. This crisis is relatively recent, escalating over the last 100 years in parallel with colonization and related social change, resulting in the widening gap of social inequities for Indigenous Peoples [9,10]. It is critical to advance meaningful youth involvement in addressing the challenge of suicide because it is very clear that solely medicalized or clinical approaches can be ineffective. Indigenous-led approaches, such as the development of the National Inuit Suicide Prevention Strategy by Inuit Tapiriit Kanatami [11], demonstrate [12] that a multifaceted approach that also addresses colonial, historical, and ongoing social inequities is needed. Youth engagement in these efforts for suicide prevention is important as a means to address the large social impacts of suicide loss experienced by many of these youth.

### 1.1. Suicide Prevention and Mental Wellness with and by Circumpolar Youth 

The Sustainable Development Working Group (SDWG) of the Arctic Council has focused on collaborative ways to address suicide and mental wellbeing across the Arctic [11]. The Arctic Council is comprised of 8 Member States, and 6 Permanent Participants who represent Indigenous Peoples of the Arctic. While recognizing the heterogenous cultures, knowledges, and strengths of these regions and advocacy groups, the SDWG has drawn from the sharing of knowledges and the collective action across these groups to facilitate a number of successful activities.

For example, under the Canadian chairmanship (2013–2015) of the Arctic Council, the SDWG advanced The Evidence-Base for Promoting Mental Wellness and Resilience to Address Suicide in Circumpolar Communities, focused on community-based initiatives and establishing promising practices for suicide prevention in Indigenous circumpolar contexts [13]. Under the US chairmanship (2015–2017), the RISING SUN initiative focused on creating metrics for suicidal behaviours, key correlates, and outcomes across Arctic States [11].

In March 2017, at the last of the three workshops held under RISING SUN, the Inuit Circumpolar Council (ICC) hosted focus groups with the goal of exploring how the 25 suicide prevention indicators identified through RISING SUN align with existing circumpolar suicide prevention strategies. Focus group participants highlighted the need to use Indigenous knowledge, strengths-based approaches, and enhance community involvement in decision-making for all aspects of suicide prevention, including research. In particular, there was a call for increasing youth engagement in future activities of the SDWG, particularly those related to suicide prevention and life promotion [14]. 

The response to these findings was the development of Project CREATeS (Circumpolar Resilience, Engagement and Action Through Story), which continued the work of the SDWG through Finland’s Chairmanship of the Arctic Council. Project CREATeS was designed to engage circumpolar youth in suicide prevention, as well as to allow youth to connect across Arctic States and Permanent Participant organizations through their participation in digital storytelling and sharing circles [3]. Through the creation of a culturally safe space for youth to talk and create stories out of their experiences, youth participants stimulated dialogue about strengths-based actions they would like to take towards suicide prevention. One of the aims that emerged through this dialogue was the intention to sustain the circumpolar youth network established through Project CREATeS. However, there are barriers to physically connecting youth across such wide geographies, including the cost of transportation and accommodations. Additionally, travel and large public gatherings was prohibited in response to the COVID-19 pandemic. In the remainder of this paper, we outline our approach to supporting youth engagement in suicide prevention through the creation of a virtual community of practice in youth mental wellbeing, by leveraging and adapting the Project ECHO model [15,16].

### 1.2. Building a Virtual Community of Practice Using the Project ECHO Model 

Although the idea of collective learning is longstanding, Etienne Wenger coined the term community of practice (CoP) to both describe this shared endeavor and distinguish it from other, more didactic forms of learning. A CoP is a group of people “who share a concern, a set of problems, or a passion about a topic, and who deepen their knowledge and expertise in this area by interacting on an ongoing basis” [17]. There are three core features of a CoP: domain, community, and practice. Members of a CoP do not just come together as a network, they share an interest and commitment in an identified *domain*, and collectively value their shared expertise. The *community* aspect refers to how knowledge is created and shared among the group through their interactions. *Practice* is also necessary within a CoP; members move beyond shared interests to co-develop shared practices, developing a shared repertoire of resources that may range from shared experiences, stories, tools, and ways of engaging within their domain.

There has been a proliferation of CoPs within healthcare, education, and other professional domains, including the emerging use of virtual technologies to support CoPs [18]. Other relevant, emerging areas including youth-led CoPs [19], and CoPs within the area of Indigenous health and wellbeing, as well as CoPs that advance community-based participation and interests. Wexler and colleagues, for example, describe building a CoP with Indigenous leaders and service providers in a rural Alaska [20]. Their CoP, Promoting Community Conversations About Research to End Suicide (PC CARES), facilitates collective learning about suicide prevention that encourages multiples knowledges and practices, including Indigenous and community-based knowledge, alongside, and engaged with, academic research practices.

Project ECHO is a model that leverages technology to create CoPs. ECHO was originally developed to support primary care providers in building capacity in the management of complex patient needs for a range of chronic health conditions in the community, using the CoP for telementoring and continuing professional development [15,21]. Although Project ECHO has been shown to improve provider and patient outcomes related to a range of chronic health conditions, including mental health [15,16], the use of the ECHO model to create a CoP or learning community ‘by and for youth’ has not been previously reported. Beginning in 2020, we developed and implemented an ECHO program specifically aimed at supporting the development of a CoP for circumpolar youth with an interest in mental wellbeing, suicide prevention, and life promotion. The major driver of this initiative was youth interest in connecting with peers across the Arctic. The following report summarizes the process we undertook in the design, adaptation, needs assessment, and implementation of ECHO to create the Arctic Youth Wellbeing Network (AYWN) [22]. Our primary questions at this stage included:

(1) Is creating a virtual CoP feasible for Arctic youth? In particular, can the ECHO model be used and adapted for this purpose?
(2) Within the domain of mental wellbeing, suicide prevention, and life promotion, what are the areas of interest for learning and knowledge sharing among youth?

## 2. Materials and Methods

We used the ECHO model, a well-known “hub-and-spoke” approach that leverages technology for building communities of practice in health care and beyond, particularly in under-resourced settings. The “hub” in the ECHO model is the central organizing and resourcing site (although this hub may be distributed across multiple settings), who also contribute expertise in the domain of the community of practice and share it with the “spoke” sites, whose members join as participants [15]. Equally important, however, is the sharing of knowledge among participants “spokes,” and with the “hub “team. These so-called “learning loops” [23] foster the domain, community, and practice aspects of a CoP. The implementation, coordination, and facilitation techniques used in the ECHO model, which are described further below, support this exchange of knowledge and help shape new practices that emerge from the community. To support the development of the AYWN, we drew upon our previous experiences implementing the ECHO Ontario Mental Health (ECHO-ONMH) Program at the Centre for Addiction and Mental Health (CAMH) in Toronto, Ontario, Canada [24], which is a well-established national and provincial program. We also adapted the model based on engagement with the Advisory group and feedback from the needs assessment conducted with participants, which is the focus for this paper.

### 2.1. Advisory Group 

This group was comprised of previous participants and organizers of Project CREATeS, as well as a more recent SDWG initiative known as Local to Global. The group also included Permanent Participant members, specifically the Inuit Circumpolar Council, (ICC), and Gwich’in Council International (GCI). There was also participation by youth, particularly through a new partnership with the Arctic Youth Network (AYN), a youth-founded and youth-led non-profit organization. The Advisory Group met virtually on a weekly basis in the three months of planning for and implementing the virtual AYWN, and then bi-weekly after the launch. The Advisory Group was formed to develop strategies to meet youth goals of maintaining and growing collaboration among Arctic youth in the area of suicide prevention and life promotion. The ECHO model was suggested as a core method of structuring this collaborative and of achieving the learning goals of youth, and was endorsed by the Advisory. The Advisory Group was critical in supporting the adaptation of core elements of the ECHO model to meet the interests, values and preferences of the youth, particularly Indigenous youth living in the Arctic.

### 2.2. Participant Recruitment

Youth were recruited through the Advisory Group, with invitations sent to all Permanent Participant organizations of the Arctic Council, as well as advertising through the Arctic Youth Network on their website, Facebook, and Twitter accounts. In order to register, youth met the following inclusion criteria: (1) Between 16 and 30 years of age; and, (2) Born in and/ or living in a circumpolar area. Youth who met inclusion criteria registered for the AYWN through an online REDCap survey. REDCap is a secure, HIPPA-compliant web application for building and managing online surveys and databases. The registration process included asking participants to provide demographic details, contact information, and solicited preferences about dates and timing of ECHO sessions, and preferred language of participation.

### 2.3. Needs Assessment Survey

Furthermore, part of the registration process, participants completed an assessment of their learning interests in order to ensure that the domain of learning in the CoP was focused on participant interest and learning needs. This needs assessment is an important step in ECHO implementation [24]. This brief survey asked participants to rate their current level of interest across a range of topics related to mental health and wellbeing, using sliders that can be set anywhere from 0 (not at all interested) to 100 (very interested). Participants were then provided with the same list of items and asked to rate their current level of knowledge in the same topic areas, from 0 (not at all knowledgeable) to 100 (very knowledgeable). They were also provided with a free-text option to indicate any additional areas of interest. The list of items included in the survey were generated by reviewing themes that youth identified as important through Project CREATeS focus groups with participating youth [3] and through input from the Advisory Group. Specific items are listed below in the results section.

### 2.4. Research Ethics Review

The protocol for this project was reviewed by the Institutional Review Board at the Centre for Addiction and Mental Health (CAMH), in Toronto, Ontario, Canada; ethics approval was waived because the AYWN was deemed to be an educational activity. We included information and consent statements on the survey explaining the purposes of the needs assessment survey, that results would be used for developing the AYWN program, and may be anonymized and presented in aggregate for reports, presentations, or publication.

### 2.5. Data Analysis

The results of the needs assessment were analyzed using descriptive statistics [25]. Below we also identify aspects of the ECHO model that were adapted to meet the unique needs of this CoP. We also include a description of sessions, with example excerpts of youth contributions to knowledge sharing. To select exemplary quotes by participants we employed directed qualitative analysis [26], reviewing audio of ECHO sessions in order to identify exemplary participant quotes to demonstrate focal points of knowledge sharing that were characteristic of the topics for particular sessions.

## 3. Results

**Figure 1:**
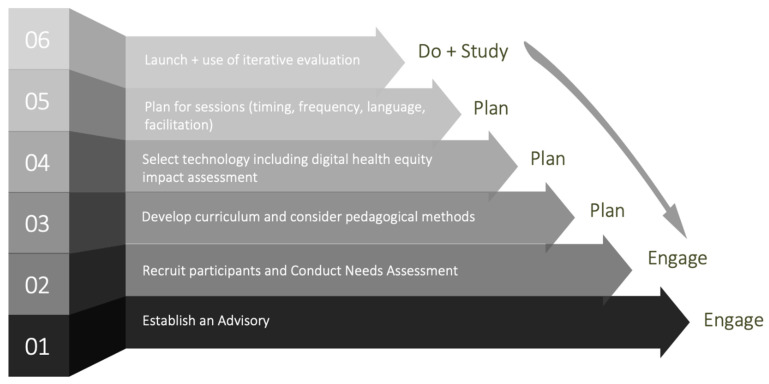
Steps taken to develop the Arctic Youth Wellbeing Network using the ECHO model.

We report on the results of our needs assessment within the context of our development and adaptation of the Project ECHO model to form the virtual AYWN. Below we describe the dimensions of the ECHO model and their application to the AYWN, including a description of adaptions of the ECHO model (See Figure 1).

### 3.1. Establishing an Advisory Group

Although it is not a defining feature of the ECHO model, we knew it was essential to have an Advisory Group rooted in community and representing circumpolar Indigenous Peoples. This group actively participated in all aspects of planning for the AYWN, meeting initially weekly and then bi-weekly over the course of planning and initiating the AYWN. It included membership from the Arctic Youth Network, Inuit Circumpolar Council International, Gwich’in Council International, Crown Indigenous Relations and Northern Affairs Canada, CAMH, University of Toronto, and University of Southern Denmark. The Advisory Group also had youth members who were suggested by the aforementioned organizations. We had opportunities throughout the project to seek further input and collaboration from other Permanent Participant members of the Arctic Council.

### 3.2. Recruitment of Participants in the CoP

37 participants registered for the AYWN. Participation was by word-of-mouth, invitation, through communications to Permanent Participant organizations, and through advertising on social media. As is typical in the ECHO model, and characteristic of a CoP, participation is constrained by how the community is defined (see inclusion criteria, above): Arctic and Arctic Indigenous youth, aged 16 to 31 years, and by their common interest in the identified CoP domain: wellbeing, mental health, suicide prevention, etc. Participant demographic characteristics are summarized in Table 1. Participants primarily resided in Canada (*n* = 17), followed by Greenland (*n* = 11), and the United States (*n* = 4). The majority identified as female (*n* = 21). The majority also identified as Indigenous (*n* = 23).

### 3.3. Conducting a Needs Assessment

An early activity of the Advisory Group was to develop a needs assessment. Although circumpolar youth engaged in a previous project of the SDWG had indicated their interest in remaining connected to youth from across circumpolar areas and continuing to develop youth-led priorities for mental wellbeing and suicide prevention, it was not clear what the specific focus or content of these activities should be. Based upon previous input from youth [3], and expertise and knowledges contributed by the Advisory Group, we generated a list of potential areas of focus related to wellbeing, mental health, suicide prevention, culture, Indigenous knowledge, and community. Youth who completed the survey were asked to rate their *interest* in learning about each topic on a scale of 0 to 100, with 0 indicating no interest in the topic and 100 indicating significant interest. Responses are summarized in Table 2. The top five topics in order of the highest mean interest scores were: suicide prevention (mean rating 87.5%); resilience (86.6%); cultural approaches to mental wellbeing (86.2%); leadership development (85.4%); and, language-based approaches to mental wellbeing (82.6%). They were also asked about their current knowledge in each of the same areas to gauge their *knowledge* and *self-efficacy* in these areas at the outset of the network. Self-efficacy and knowledge scores were also rated on a scale of 0 to 100, with 0 indicating “not knowledgeable” and 100 indicating “Knowledgeable.” Responses are summarized in Table 2. The top five topics based upon lowest self-rated efficacy or knowledge by mean rating were: social activism and advocacy (44.5%); government and policy (44.9%); social media and mental wellbeing (48.3%); language-based approaches to mental wellbeing (48.7%); leadership development (51.4%). 

### 3.4. Developing a Curriculum

The curriculum was codesigned with the Advisory Group, utilizing results from the needs assessment, and based upon principles of curriculum development [27]. Because the topics of interest were ranked differently than areas of knowledge or self-efficacy, we looked at the delta between the two ratings and prioritized topics with the greatest interest and lowest knowledge/self-efficacy (i.e., with the greatest delta between self-reported scores). This resulted in the following priorities: cultural approaches to mental wellbeing (delta of 34.6); language-based approaches to mental wellbeing (33.9); resilience (33.0); government and policy (28.8); and suicide prevention (25.8). We also identified several relevant skills of interest to the group, which we aimed to weave throughout sessions: leadership development (34.0); social activism and advocacy (30.5); and project management and facilitation skills (27.1). The Advisory Group helped to identify guest speakers with expertise in these key areas. Table 3 lists sample session topics, speakers, and the way each topic fit in with identified learning needs.

### 3.5. Technology

One of the tenets of the ECHO model is leveraging technology, in this case videoconferencing software, to create a CoP [15]. We used Zoom communications technology because it is broadly accessible, is telephone compatible (i.e., with or without video), and uses minimal internet bandwidth. It also meets standards of security, and additional measures—for example, passwords required to join the sessions—can also be utilized. We offered technical support and a practice session with participants prior to the first session. The choice to use videoconferencing software did create an implicit set of inclusion/exclusion criteria, specifically related to reliable access to internet, mobile data, and necessary hardware, as well as digital literacy more broadly [28]. We address the implications of digital tools on equity within a CoP in the discussion below.

### 3.6. Facilitation of the CoP

Our hub differed somewhat from the typical ECHO model in that hub members were dispersed across sites in Canada, Greenland, and Denmark, and, similarly, our expert guest speakers originated from across the circumpolar region and represented a range of professions (university, community-based, health, artists). This more expansive hub membership was critical to the aims of the AYWN, which were to foster exchange and sharing across the Arctic. For this reason, facilitation of each session was also shared across hub members, including youth leads.

Because ECHO sessions necessarily involve multiple participants, they are carefully structured using a predetermined agenda, which the facilitator uses to guide stages of the session and group discussions. The ECHO model relies upon three principles of facilitation (as well as related techniques) to encourage effective capacity building among participants. First, the length of didactic presentations are brief in comparison to the time allocated for group participation. Second, participants (“spokes”) are invited to contribute and to share their knowledge of the session topics before hub members do so.; Third, all participants use built-in features of the videoconferencing software (e.g., the ‘raised hand’ alert, chat functionality, and mute/unmute functions) to improve communication. Additionally, during each session a hub member is responsible for managing these features to ensure the facilitator can focus on session content. 

We added two additional facilitation techniques to the AYWN: we used a sharing circle format [29] where participants were given the opportunity to speak or to pass (by using the zoom function to highlight their screen) instead of using raised hands; and, we invited each speaker to start the session with any ceremony or practice that was meaningful to them (e.g., prayer, drumming, song, story) and we finished each session with a guided relaxation or reflective exercise, often led by a youth member of our hub team. We intended these facilitation techniques to strengthen the CoP by ensuring that all felt they had space to participate, by valuing the diverse practices and knowledge from across participants, and by marking our time together as special and as a time to be honored.

### 3.7. Session Structure

In the planning stage, we had to consider elements that would shape the session structure (See Box 1). The ECHO Model does not require a minimum number of sessions, regular schedule, or frequency of sessions, although our previous experience indicates it is important to find a time that is a best-fit across the CoP to reduce barriers to participation, and that infrequent or irregularly scheduled sessions can diminish community cohesion. While sessions can be 1 h in length, this does not allow for as much interactivity, while sessions that are too long can pose a barrier to participation. In the needs assessment, we asked participants to identify the best days, times of day, and frequency of sessions to encourage their involvement. We were somewhat constrained in determining an optimal time of day due to of the broad geographic distribution (up to 12 h across time zones) among participants. We selected monthly sessions of 1.5 h in length, on the day chosen with the highest frequency (Friday), for our first cycle of the AYWN. We also asked participants and the Advisory Group for their preferences and advice on the language/s used during the sessions. Most participants had some familiarity with English and preferred to have a shared language for sessions. We also offered interpretation services if desired and translated written materials and communications into the languages of participants.

Our sessions were structured with a facilitation agenda (see Box 1) to ensure efficiency and maximize participation. As an additional element, we encouraged participants to introduce themselves at the beginning of each session to help build a shared sense of community among participants. In addition, we reaffirmed principles to enhance psychological safety within each session, including reminders about confidentiality, and that any of us may experience emotion. We invited members to reach out to a specified person in the event that they wanted support, asked them to notify the hub if they planned to leave the session, and requested them not to share details of a traumatic event (including details of suicide loss or attempts) in an effort to reduce the risk of distress due to exposure to detailed traumatic descriptions. We also discussed with them the use of inclusive and suicide-safe language and the role of stigma in mental wellbeing. Participants were encouraged to share with the group or decline as desired, as well as to seek permission before offering feedback or suggestions to other members.

Each session began with a 20–30 min presentation by either a hub member or invited guest speaker on a topic aligned with the learning priorities identified by the needs assessment (see Table 3). These presentations were followed by a sharing circle among participants, or an interactive or skills-based activity.

Box 1 **Session structure for the AYWN.**

Session Structure—90 min

Prior to sessions consider: Timing of sessions (time of day and week, length, frequency); language of sessions and language interpretation when necessary. Offer technology practice and support for participants, and designate role for someone to operate the technology during sessions. Develop a facilitation agenda/guide.

1. Welcome and introductions (5–10 min)
2. Review of agreements to enhance psychological safety: confidentiality; seeking emotional support; use of inclusive and suicide-safe language; refraining from sharing traumatic details (5 min)
3. Opening ritual by speaker (e.g., prayer, song, drumming, etc.) (5 min)
4. Presentation based upon co designed curriculum (20–30 min)

### 3.8. Learning and Engagement Strategies

One of the areas of greatest ongoing development within the AYWN is in the pedagogical methods used to enable the CoP to flourish. There was a great deal of mutual reinforcement between the topics of interest identified by participants (i.e., through the needs assessment) and the speakers and presentations which were chosen to align with these areas of interest to the CoP. The emergent sharing of knowledge by participants, also both contributed to meeting learning interest, and also sparked new areas of interest for exploration. Facilitation of all of these facets ensured knowledge sharing across these modalities to create what are known as ‘learning loops.’ Within ECHO there is the aspiration that “all teach, all learn” [23], and the facilitation and turn-taking of the sessions reinforces this democratizing process of learning. Table 3 includes some exemplary quotes taken from sharing circles within each session. The quotes highlighted the way that the topic of the session is built upon through these learning loops. 

Some of the pedagogical methods associated with the ECHO model were not appropriate for this CoP due to their original focus on continuing educational development for healthcare professionals. We incorporated methods of Indigenous education and knowledge sharing, such as the use of sharing circles, storytelling, and practices for wellbeing drawn from across Indigenous circumpolar traditions and best practices. We also introduced elements that we felt would appeal to our youth participants, including the use of arts-based methods. An additional pedagogical area emerged during this first cycle, and that is youth interest in skills-based learning. Several sessions were very active and allowed youth to practice and share skills, such as learning about collage.

## 4. Discussion

As we discovered previously through the rich insights generated by youth participants in Project CREATeS, strengths-based approaches are not effective without the active engagement of youth in their design [3]. This is true for Arctic Indigenous youth not only because they are disproportionately impacted by suicide, but also because one of the factors associated with the rise in suicide among Indigenous youth globally, including in the Arctic, are the ongoing inequities associated with colonization [9,10,12]. It is inconceivable that these trends will be reversed through more of the same paternalism, disempowerment, and silencing. Instead, interventions must be strengths-based, create space for youth ideas, and support action by youth.

The impetus for the creation of the AYWN came from the participants themselves, who also co-designed the initiative. Youth derived a sense of belonging and encouragement in Project CREATeS by connecting with youth from across circumpolar States and Permanent Participants. The AYWN was intended to sustain and grow that network.. The needs assessment completed by youth participants helped us to identify existing gaps between areas of interest and self-reported knowledge. The session topics identified to address these gaps included cultural approaches to mental wellbeing, language-based approaches to mental wellbeing, resilience, government and policy, suicide prevention leadership development, social activism and advocacy, and project management and facilitation skills.

CoP offered a model that brought together both the need for continued connection (community) and for learning (practice) to support knowledge sharing about suicide prevention and mental wellbeing, the domain of interest of these youth. More specifically, the ECHO model enabled this CoP to come together virtually. We adapted the core aspects of ECHO—its use of videoconferencing technology, defined session structures, and facilitation techniques –to meet the specific needs of a diverse community of circumpolar youth. This is the first reported use of the ECHO model involving youth, and specifically in the context of Indigenous knowledge sharing. Our success in planning and implementing AYWN demonstrates the feasibility of using the ECHO model to support youth to form CoPs.

The development of this CoP also benefited from additional adaptations to meet the learning needs of these youth. First, an Advisory Group consisting of Arctic, Indigenous, and youth stakeholders was essential to every element through the planning, participant recruitment, needs assessment and curriculum development phases of this project. The facilitation techniques also benefited from the inclusion of additional roles, including technology support, and the involvement of youth on the hub team and in leadership roles. Probably the most important innovation, and one that is very much ongoing, was considering the learning preferences and pedagogical methods that best suit this diverse community. Unlike the prototypical health professions learners on other ECHOs, youth showed a preference for a wide range of creative methods for learning, and they valued skills-based activities.

Indigenous knowledges and pedagogical strategies were also essential to AYWN. The speakers and youth participants shared knowledge through storytelling, song, drumming, and across generations. The introduction of a sharing circle as a method of facilitation aligned with these pedagogical strategies and resonated with participants. Opening and closing sessions by sharing local and cultural practices was intended to mark the space as a special place of sharing and respect.

Some immediate limitations were the limited use of Indigenous languages as we all defaulted to a common language of English. On one hand, this allowed participants from diverse linguistic and cultural groups to engage with each other, while on the other, language was identified as an important driver of mental wellbeing. In addition, although the aim was to sustain and grow a network of Indigenous youth across the circumpolar region, we were most successful at recruiting Inuit and Gwich’in youth living in Canada, and Greenlandic Inuit living in Greenland and Denmark, with less participation from youth residing in other Arctic countries and/or belonging to other Indigenous cultural groups. This is likely related to the complement of members who were active on the Advisory Group, and even to the States and Permanent Participants who were engaged in this initiative at the Arctic Council level.

Another limitation is that this is a first cycle of the AYWN, and thus still has a relatively small membership which will require time to deepen and grow. Because CoPs can change as their membership changes, some of our current processes may not remain relevant to the CoP as it evolves. Similarly, the needs assessment and curriculum will need to be iteratively assessed and redeveloped. However, our experience has been that the core considerations and elements for planning an ECHO to support a CoP among Indigenous youth remain applicable and can guide future implementation of similar CoPs using the ECHO model.

Another area of limitation is reliance on access to certain technologies to both facilitate and participate in ECHO programming. Videoconferencing technology enabled this community to form across numerous Arctic states and time zones. However, barriers to accessing these technologies may exacerbate or create further inequities [28]. For example, access to high-speed internet, necessary hardware, and the private space to participate, may create barriers for some youth and actually lead to exclusion. A more subtle issue is the unresolved tension between the imperatives of technology, which tends to erase local specificity and to ‘zoom over’ place, and Indigenous ways of knowing that are particularly relevant to mental wellbeing. Land, for example, is increasingly recognized as critical to wellbeing and identity [30]. How will this be accounted for when technology is used to support wellbeing? So while knowledge can be shared through this medium, there is also the awareness that it may be changed altogether.

Evaluation of the implementation of AYWN, including a focus on concepts like sustainability and growth as the CoP matures, will be a critical priority. Ongoing outreach and recruitment will hopefully lead to meaningful growth of the community. Other necessary considerations include evaluation of youth experience and their satisfaction with the model, accruing evidence of the learning outcomes of the youth, and whether AYWN meets the learning interests identified through the needs assessment. Addressing the limitations identified above is also a priority, in particular assessing AYWN for any equity impacts, specifically those related to digital equity.

## 5. Conclusions

A CoP can support youth-identified learning needs in the areas of suicide prevention and life promotion. The ECHO model was utilized to create a CoP across the circumpolar region, supporting Indigenous youth to come together to advance their own goals and interests. Adaptations to the model were required to ensure its success with the involvement of Indigenous youth participants, for whom ECHO was not originally designed. If the early success and development of AYWN leads to ongoing growth and sustainability of this community it could be a powerful method to support youth to meet their goals for community, connection, and knowledge sharing across circumpolar States and Arctic Council Permanent Participants. In order to do so, however, we need to further evaluate the outcomes of this model. Future growth of this community of practice will also require ever increasing youth leadership, and the hope is that the AYWN community itself will provide a venue for nurturing youth leadership.

## Figures and Tables

**Table 1:** Characteristics of AYWN Participants.

Characteristic	Number of Participants	Percent of Participants (%)
Total participants	37	
Age group		
16 to 20	5	13.5
21 to 26	18	48.6
27 to 31	13	35.1
No response	1	2.7
Birth country		
Canada	17	45.9
Iceland	1	2.7
USA	4	10.8
Sweden	1	2.7
Russia	1	2.7
Greenland	11	29.7
Norway	1	2.7
Greece	1	2.7
Gender identity		
Male	7	18.9
Female	21	56.7
Gender fluid	1	2.7
Not specified	8	21.6
Identity		
Indigenous	23	62.1
Non-Indigenous	8	21.6
Not specified	6	16.2
Representative organization		
Gwi’chin Council International	2	5.4
Inuit Circumpolar Council	17	45.9
Saami Council	2	5.4

**Table 2:** Results of participant needs assessment—mean self-reported interest and knowledge scores.

Topic	Mean Self-Reported Interest Score, 0 = Not Interested, 100 = Extremely Interested	Mean Self-Reported Knowledge Score, 0 = not Knowledgeable, 100 = Knowledgeable	Delta between Interest and Knowledge by Topic
Understanding mental wellbeing			
Suicide prevention	87.5	61.7	25.8
Psychological trauma	75.7	55.6	20.1
Substance use	74.1	59.4	14.7
Resilience	86.6	53.6	33.0
Childhood experiences	80.9	60.3	20.6
Promoting mental wellbeing			
Cultural approaches	86.2	51.6	34.6
Language approaches	82.6	48.7	33.9
Arts	74.3	55.6	18.7
Sports	63.3	61.2	2.1
Social and political context			
Social media	68.8	48.3	20.5
Climate change	77.2	57.1	20.1
Government and policy	73.7	44.9	28.8
Skills development			
Project management and facilitation skills	78.9	51.8	27.1
Leadership development	85.4	51.4	34.0
Social activism and advocacy	75.0	44.5	30.5
Digital tools and technology	75.1	56.8	18.3

**Table 3:** Session and speaker examples.

Session Topic and Associated Learning Priorities from Needs Assessment	Guest Speaker	Learning and Engagement Strategies	Examples from Sharing Circle
Storytelling and Indigenous science - cultural approaches to mental wellbeing - resilience	Jocelyn Joe-Strack Daqualama, Champagne and Aishihik First Nation, Canada	Storytelling Drumming Sharing circle	“I’m still learning our stories. I love hearing old stories…it really makes me want to learn more.” “I always think of the stories as a gateway culture thing. Once you start learning the stories you get hungry for more.” “We also have stories like these in Greenland about how you should respect nature and especially about food. You can pick up from the water but you have to be careful that you don’t take too much fish from the water and you have to use it well. In that way, I think spirituality can be powerful. But you also have to not just tell it to our youth, but also they have to understand the meaning of the stories. I think we could use [storytelling] much more in Greenland. In a modern way, like make stories with films much more.”
Iñupiat cultural values and family systems - cultural and language-based approaches to mental wellbeing - childhood experiences	Maamaq Linda Joule, Iñupiat, Northwest Alaska	Elder teachings Sharing circle	“The way knowledge is shared is through the Elders. I think that’s so beautiful because we don’t just write it down in a book…we share knowledge through the Elders. I feel so connected to my culture when I take to an Elder.” “I think connection of all ages, but especially Elders is so important. I commercially fish with my family. That’s a really important time for all of us, because you have the little kids, and my grandmother all on the boat together when it’s calm enough for them. It shows a deeper meaning. I’ve been [away] for two years now, and it’s a lot of Western ideology. Accolades are at the individual level and it’s just not as fulfilling.”
Suicide prevention by and for Sámi- suicide prevention - cultural and language-based approaches to mental wellbeing - government and policy	Jon Petter Stoor Laeves Sámi, Sweden	Music, video Sámi cultural knowledge	“When I’m with my family and we’re just on the land. We call it salmon berries because it looks like salmon eggs to us. And we’re cutting fish and filling the smoke house. That’s like the most me I ever feel. So I think it’s taking that context away is a missed opportunity for looking at mental health and how food plays in so much with mental health.” “When I think of the importance of considering culture in suicide prevention, to me it just reflects how Indigenous people already live their lives. We’re very holistic people and we have a holistic worldview. When you take this very one critical component out of suicide prevention or healthcare, you’re no longer looking at it in a holistic way or treating it in a holistic way. And so for me it’s really important because it shows this wholeness of the person.”
Animal teachings and resilience - resilience - cultural and language-based approaches to mental wellbeing	Lisa Boivin, Deninu K’ue, First Nation Canada	Collage Dene animal teachings	“The reindeer symbolizes wellness. Whenever I see a herd of reindeer of just one reindeer, I know life is going to be good. Whenever I eat reindeer meat, life is good. I know I’ll be healthy. I know I’ll have energy to do physical labor.I know I’ll have the energy to keep my mental well being at very good, legendary levels.” “One thing that I’ve noticed in my culture is that it’s really important to hold your end of the deal and when I think about the reindeer and what other Arctic communities, what their teachings of the reindeer or caribou, it’s been really important for Arctic communities to be diplomatic and to hold their end of the bargain or deal.”
Music, colonization and advocacy - resilience - cultural and language-based approaches to mental wellbeing - advocacy	Karina Møller, GreenlandicAlaska	Discussion Sharing circle	“As Greenlanders, we think about us, immediately. How can I use my passion and my gifts, how can I use it to help us? We have to acknowledge, and take care, and treasure that way of existing as a society.” “We cannot accept any people within the educational system that speak down to the students. It’s not acceptable because it has consequences for the young people.”
Building bridges between traditional and modern Sámi music - resilience - advocacy	DJ iDJa Markus J. ThonhaugenSámi, Norway	Electronic music Joik Digital art	“It’s definitely a political act to exist as an Indigenous person today, unfortunately.” “I’m seeing this positive trend where more and more people are claiming their heritage and they’re doing it with bravado.” “Helping others with their creative outlets…and ensuring that other people understand what emotions are and how to express them in healthy ways, that also helps me.”
